# Cerebral protection by remote ischemic post-conditioning in patients with ischemic stroke: A systematic review and meta-analysis of randomized controlled trials

**DOI:** 10.3389/fneur.2022.905400

**Published:** 2022-09-21

**Authors:** Meng Lu, Yujiao Wang, Xin Yin, Yuanyuan Li, Hongyan Li

**Affiliations:** ^1^Department of Nursing, The First Bethune Hospital of Jilin University, Changchun, China; ^2^Department of Neurology, The First Bethune Hospital of Jilin University, Changchun, China

**Keywords:** stroke, ischemia, ischemic post-conditioning, systematic review, meta-analysis

## Abstract

**Background:**

There is evidence that remote limb ischemic postconditioning (RIPostC) can reduce ischemia-reperfusion injury (IRI) and improve the prognosis of patients with ischemic stroke. However, so far, only few relevant clinical studies have been conducted. Therefore, we carried out a meta-analysis of eligible randomized controlled trials to compare the RIPostC group with a control group (no intervention or sham surgery) in patients with ischemic stroke.

**Methods:**

Four English-language publication databases, PubMed, Cochrane, Embase, and Web of Science, were systematically searched up to March 2022. The data were analyzed using Review Manager fixed-effects and random-effects models.

**Results:**

A total of 12 studies were included, and 11 of those were analyzed quantitatively. Compared to controls, The RIPostC group showed significantly reduced NIHHS scores in patients with ischemic stroke, (MD: −1.09, 95% confidence interval [CI]: −1.60, −0.57, *P* < 0.0001) and improved patients' Montreal Cognitive Assessment (MoCA) scores, (MD: 1.89, 95% CI: 0.78, 3.00, *P* = 0.0009), Our results showed that RIPostC is safe, (RR = 0.81, 95%CI: 0.61, 1.08, *P* = 0.15).

**Conclusion:**

Our meta-analysis showed that RIPostC is safe and effective and has a positive cerebral protective effect in patients with ischemic stroke, which is safe and effective, and future large-sample, multicenter trials are needed to validate the cerebral protective effect of RIPostC.

## Background

Ischemic stroke is a highly morbid and disabling disease. About two-thirds of patients are left with sequelae including functional impairment of varying degrees (1). Early revascularization therapy such as intravenous thrombolysis and mechanical thrombectomy is an effective treatment for ischemic stroke, but this treatment has a strict time window beyond which the probability of adverse effects will increase, the salvage rate for ischemic penumbra will greatly reduce, and the risk of bleeding will outweigh the therapeutic benefit (2). In recent years, several studies have aimed to explore new neuroprotective strategies. However, few have been successfully translated from basic research to clinical application.

The phenomenon of ischemic preconditioning was first identified in the heart. Ischemic preconditioning provides hope to the study of neuroprotective measures. In 1986, the American scholar Murry performed four ischemia-reperfusion sessions on the anterior descending branch before preparing an infarct model and found that it could lead to a reduction in infarct size (3). In several subsequent studies, it has been confirmed that ischemic preconditioning can reduce myocardial infarct size and coronary vascular injury and improve the clinical prognosis of patients with myocardial infarction (4, 5). Kitagawa et al. showed that ischemic preconditioning of the gerbil brain prior to ischemia had a protective effect on the post-ischemic brain and reduced neuronal death in the C1 region of the hippocampus (6). However, ischemic preconditioning requires intervention before the onset of an ischemic event, which is difficult to achieve in clinical practice because we may not be able to anticipate sudden events.

In 2006, Zhao et al. (7) first found that ischemic post-conditioning attenuated ischemia-reperfusion injury (IRI) after cerebral reperfusion in a rat model of permanent middle cerebral artery occlusion and transient bilateral carotid artery occlusion. Similar to ischemic preconditioning, ischemic post-conditioning is an endogenous mechanism that stimulates endogenous protective mechanisms in the body to reduce IRI to critical vital organs through repeated, transient, non-lethal ischemic treatment of the body. Vinten-Johansen et al. found that post-treatment not only produced similar results to pretreatment, but that post-conditioning reduced infarct size, attenuated vascular dysfunction, and reduced neutrophil accumulation after prolonged reperfusion (8–10). Several studies have confirmed this finding (11–13). Studies have shown that ischemic post-conditioning can inhibit free radical production and initiation of apoptosis during ischemic reperfusion and increase superoxide dismutase and catalase activities in brain tissue (7, 14). Reactive oxygen and nitrogen (ROS/RNS) also play an important role in the mechanism of action of ischemic post-conditioning (15). Although the protective mechanisms are not yet fully elucidated, the protective effect of ischemic post-conditioning on the brain has been confirmed in several studies.

RIPostC is safer than local post-conditioning. RIPostC refers to ischemic post-treatment of non-life-critical organs such as skeletal muscles of the arms and legs to mediate the body's endogenous protective mechanisms, and is a complex, systemic phenomenon (16). RIPostC was first used 30 years ago in patients who suffered a heart attack (17, 18), and it can exert a protective effect on the myocardium through a complex signal transduction including neuronal and humoral (19). Similarly, RIPostC not only protects the myocardium from ischemic injury, but also has a similar protective effect on the brain (20). However, most of these studies have focused on animal models and have achieved ischemic post-conditioning by intermittent occlusion of the carotid and cerebral arteries, which is ethically not possible in humans, as it is associated with high risk. Several clinical studies have investigated the protective effect of post-treatment of RIPostC in patients with ischemic cerebrovascular disease, but the number of available studies is small and the sample size of individual studies is very limited to draw definitive conclusions. Therefore, a systematic evaluation in conjunction with published randomized controlled trials (RCTs) is needed to assess whether RIPostC improves the prognosis and reduces the degree of neurological deficits in patients with ischemic cerebrovascular disease.

## Materials and methods

We followed the Cochrane Handbook of Systematic Reviews of Interventions for this systematic review and meta-analysis. We reported the meta-analysis according to the Preferred Reporting Items for Systematic Reviews and Meta-Analyses (PRISMA) statement (21).

### Selection of study subjects

Inclusion criteria: (1) Patients of any age, sex, and race with ischemic cerebrovascular disease. (2) The test group received RIPostC including any site, any duration, and any pressure. The control group was a sham training group or blank control. (3) Primary outcome indicators were National Institutes of Health Stroke Scale (NIHSS) score and modified Rankin Scale (mRS). The NIHSS score is used to assess the degree of neurological deficits in stroke patients. The assessment items include patient's consciousness, eye movements, visual field, limb muscle strength and sensation, limb ataxia, speech function, cognitive and attention. The assessment scores range from 0 to 42, with higher scores indicating more severe neurological impairment. The mRs is used to measure the neurological recovery of patients after stroke, with a score range of 0–6. The higher the score, the worse the prognosis; generally, a score < 2 is associated with good prognosis. Secondary outcome indicators included Montreal cognitive assessment (MoCA) score, Barthel Index (BI) score, incidence of adverse events in both groups, and cerebral infarct volume. (4) Inclusion of all studies as RCT studies.

Exclusion criteria: articles that do not contain the above ending indicators.

### Literature screening and data extraction

A primary screening and re-screening of the literature was performed according to the inclusion criteria enjoyed in this study, and data extraction of the included literature was performed. Literature screening and data extraction were independently carried out by two investigators (LM and WY), in case of disagreement, a third investigator (LY) intervened and a consensus was reached. Data related to study characteristics, quality, and outcomes were extracted using a standardized information extraction form. We extracted the following information: (1) general information: study site, RIPostC intervention procedure, number of patients in both groups, and intervention start time; (2) primary outcome indicators: NIHSS score and mRs score; and (3) secondary outcome indicators: Barthel score, MoCA score, incidence of adverse events in both groups, and cerebral infarct volume.

### Quality evaluation

Three investigators independently evaluated the included literature and cross-checked the results using the risk of bias assessment tool for RCTs recommended in the Cochrane Handbook (5.1.0), and in case of disagreement, a third investigator arbitrated until consensus was reached. The evaluation included the following seven aspects: (1) selection bias: whether a randomized series was generated; (2) whether the allocation scheme was concealed; (3) implementation bias: whether the investigators and subjects were blinded; (4) measurement bias: whether the assessment of outcome indicators was blinded; (5) follow-up bias: whether the outcome data were complete; (6) reporting bias: whether there was selective reporting of results; and (7) whether there were other sources of bias.

### Statistical analysis

Meta-analysis was performed using Rev Man 5.3. Standardized mean difference (SMD) or mean difference (MD) were used as effect analysis statistics for continuous variables, and risk ratio (RR) or odds ratio (OR) were used as effect analysis statistics for dichotomous variables. In total of 95% confidence intervals (CI) are provided for each effect size. Heterogeneity among the included studies was analyzed by combining the chi-square test and I^2^ values and if *P* > 0.1 and I^2^ < 50%, this indicated good homogeneity and a fixed-effects model was used, but if *P* < 0.1 and I^2^ ≥ 50%, it indicated significant heterogeneity and a random-effects model was used. For all analyses, *P* < 0.05 was considered to indicate statistically significant differences.

## Results

### Literature search

**Figure 1:**
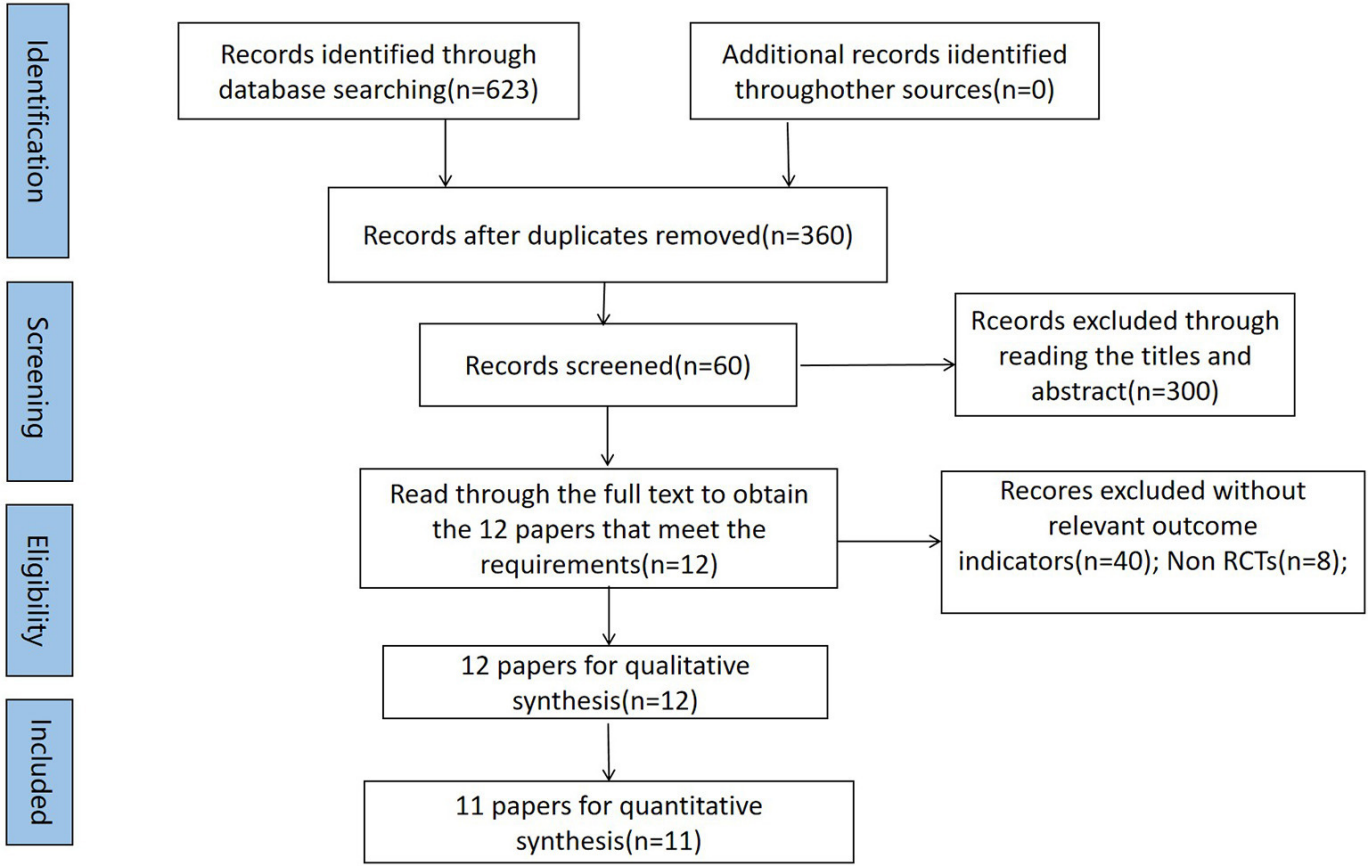
PRISMA flow diagram showing study identification and selection.

The Cochrane Library, PubMed, Web of Science, and Embase databases were searched online, and the references of the included literature were searched retrospectively with a search time frame until March 2022. Twelve eligible studies were eventually included in the systematic evaluation, and 11 studies were included in the meta-analysis. A search strategy was developed using subject terms combined with free words, and the search strategy and search flow chart are as shown in Table 1 and Figure 1 respectively.

**Table 1:** Search strategy form.

**1. Pubmed**	
#1	Search: (((((((strokes) OR (stroke)) OR (cerebrovascular accident)) OR (brain vascular accident)) OR (ischemic stroke)) OR (cerebral strokes))) OR (brain infarction) 40763
#2	Search: ((((ischemic postconditioning) OR (ischemic preconditioning)) OR (remote preconditioning)) OR (remote postconditioning)) OR (RIPostC) 12848
#3	Search: (#1) AND (#2) 1463
#4	Search: (#2) AND (#3) Filters: Clinical Trial, Randomized Controlled Trial 107
**2. Web of science**
#1	TS=((((((((strokes) OR (stroke)) OR (cerebrovascular accident)) OR (brain vascular accident)) OR (ischemic stroke)) OR (cerebral strokes))) OR (brain infarction)) 586005
#2	TS=(((((ischemic postconditioning) OR (ischemic preconditioning)) OR (remote preconditioning)) OR (remote postconditioning)) OR (RIPostC)) 16561
#3	(TS=((((((((strokes) OR (stroke)) OR (cerebrovascular accident)) OR (brain vascular accident)) OR (ischemic stroke)) OR (cerebral strokes))) OR (brain infarction))) AND TS=(((((ischemic postconditioning) OR (ischemic preconditioning)) OR (remote preconditioning)) OR (remote postconditioning)) OR (RIPostC)) 1895
#4	(TS=((((((((strokes) OR (stroke)) OR (cerebrovascular accident)) OR (brain vascular accident)) OR (ischemic stroke)) OR (cerebral strokes))) OR (brain infarction))) AND TS=(((((ischemic postconditioning) OR (ischemic preconditioning)) OR (remote preconditioning)) OR (remote postconditioning)) OR (RIPostC)) and Clinical Trial (Literature Type) 120
**3. Embase**
#1	(strokes OR stroke OR (cerebrovascular AND accident) OR (brain AND vascular AND accident) OR (ischemic AND stroke) OR (cerebral AND strokes) OR (brain AND infarction))
#2	(ischemic AND postconditioning OR (ischemic AND preconditioning) OR (remote AND preconditioning) OR (remote AND postconditioning) OR ripostc)
#3	#1 AND #2 2163
#4	#1 AND (“clinical trial”/de OR “randomized controlled trial”/de) 136
**4. Cochrane library**
#1	((((((((strokes) OR (stroke)) OR (cerebrovascular accident)) OR (brain vascular accident)) OR (ischemic stroke)) OR (cerebral strokes))) OR (brain infarction)) 80759
#2	((((ischemic postconditioning) OR (ischemic preconditioning)) OR (remote preconditioning)) OR (remote postconditioning)) OR (RIPostC) 1729
#3	(#1) AND (#2) 260

### Characteristics of included studies

A total of 12 eligible studies were included (22–33), all of which included patients with ischemic cerebrovascular disease. Eleven studies were analyzed quantitatively, and one study (33) was analyzed only qualitatively because the data could not be accurately extracted. A total of 713 patients were included, 353 in the trial group and 360 in the control group. Ten of the 12 studies were in patients with acute ischemic stroke, and the remaining two studies were in patients with atherosclerotic stenosis. Three studies reported using intravenous thrombolytic therapy (25, 29, 30), and the remaining nine studies used conservative treatment strategies. The studies were conducted in four countries: China, United Kingdom, France, and Romania. Of the 12 included studies, one study was conducted in the lower extremity (32) and the remaining 11 studies were conducted in the upper extremity. The duration of individual cycles was 5 min in all RIPostC groups, and the intervention pressures were 180–200 mmHg or greater than 20–30 mmHg systolic in all groups except the lower extremity group. Two of the 12 included studies (22, 28) used the same group of patients, so the demographics are not presented separately, and in conducting meta-analysis we will also follow a sample size. The general demographic information and study characteristics of the studies are shown in the Tables 2, 3.

**Table 2:** Research characteristics.

**Inclusion in the study**	**Sample size**		**Interventions**		**Country**	**Outcome indicator**
	**Experimental group**	**Control group**	**Experimental group**	**Control group**		
Meng et al. (22)	30	28	Patients with symptomatic intracranial atherosclerotic stenosis were subjected to 5 cycles of simultaneous ischemia in both upper arms for 5 min followed by 5 min of reperfusion twice a day for 180 days at a pressure of 200 mmHg during pressurization.	Performed sham RIPostC training with the same training duration and period as the test group, with a pressure of 30 mmHg during pressurization.	China	NIHSS
England et al. (23)	13	13	The upper extremity of the healthy side received 4 cycles of 5-min compression and 5-min relaxation of RIPostC training within 24 h after the onset of AIS, and the pressure at compression was 20 mmHg greater than the systolic pressure.	The pressure in the control group was 30 mmHg during pressurization, and other intervention procedures were the same as in the experimental group.	United Kingdom	NIHSS; BI; AE
Li et al. (24)	29	31	Four cycles of 5-min pressurization and 5-min relaxation of RIPostC training were performed on the healthy upper extremity within 72 h of AIS onset at a pressure of 20 mmHg greater than the systolic pressure for 14 days.	The pressure in the control group was 30 mmHg during pressurization, and other intervention procedures were the same as in the experimental group.	China	NIHSS; mRs; cerebral infarction volume.
Che et al. (25)	15	15	The experimental group received 1 RIPostC within 2 h after intravenous thrombolysis in patients with AIS and twice daily RIPostC starting the next day and continuing until day 7. The RIPostC was 5 min of pressurization and 5 min of relaxation with a pressure of 200 mmHg.	Received routine medical care.	China	NIHSS; BI; AE
England et al. (26)	31	29	Four 5-min cycles were performed in the healthy upper extremity within 6 h of AIS onset, with pressures of 20 mmHg greater than the systolic pressure at pressurization. The first 20 received one dose (four cycles) of RIPostC, 21–40 patients received a second RIPostC (4 cycles) 4 h after the first RIPostC, and the last 20 received twice daily for 4 days starting the day after the first RIPostC.	The control group received sham RIPostC with a cuff pressure of 30 mmHg at pressurization, and the other intervention protocols were the same as in the experimental group.	United Kingdom	NIHSS; BI
Feng et al. (27)	42	44	5 cycles of 5-min compression-relaxation RIPostC at a pressure of 200 mmHg in the acute phase of AIS on the healthy upper extremity for 6 months.	The control group did not perform any RIPostC training.	China	MoCA
Zhou et al. (28)	30	28	Patients with intracranial atherosclerotic stenosis underwent one 5-min ischemia and 5-min reperfusion RIPostC session alternating between both upper extremities bilaterally for 300 days.	No description	China	MoCA
An et al. (29)	34	34	The RIPostC intervention was started within 3 h after intravenous thrombolysis of the AIS and twice a day during hospitalization, with each cycle consisting of 5 min of ischemia and 3 min of reperfusion at a pressure of 180 mmHg.	Routine care medical measures without any RIPostC training.	China	mRs; AE
He et al. (30)	24	25	Two sessions of RIPostC training 6–24 h after AIS intravenous thrombolysis, each with 5 min of ischemia-reperfusion to the healthy upper extremity at a pressure of 200 mmHg.	The pressure in the control group was 60 mmHg at pressurization, and other interventions were the same as in the experimental group.	China	NIHS; mRs; AE
Li et al. (31)	24	24	Patients with AIS underwent 4 cycles of RIPostC training on the non-paralyzed side of the arm at a pressure of 20–30 mmHg above systolic pressure for 7 consecutive days within 3 days of the onset of AIS.	The control group received sham RIPostC training (cuff inflation to 30 mmHg).	China	MoCA; AE
Pico et al. (32)	93	95	Within 6 h of the AIS onset, 4 cycles of RIPostC were performed on the unilateral lower extremity, each cycle comprising 5 min ischemia and 5 min reperfusion with a pressure of 110 mmHg above the systolic pressure.	Patients in the control group had the cuff placed around the thigh on the unaffected side for 40 min without any pressure.	France	NIHSS; mRs; AE; Cerebral infarction volume
Poalelungi et al. (33)	18	22	Within 24 h of AIS onset, three 3-min ischemia sessions followed by 5-min reperfusions were performed twice daily for 5 consecutive days in the healthy upper extremity, with pressures of 20 mmHg greater than the systolic pressure during pressurization.	The pressure at pressurization was 30 mmHg, and the rest was the same as the experimental group.	Romania	Cerebral infarction volume

NIHSS, National Institute of Health stroke scale; mRS, modified Rankin scale; BI, Barthel Index; MoCA, Montreal Cognitive Assessment; AE, incidence of RIPostC adverse events; AIS, acute ischemic stroke.

**Table 3:** Demographic information.

	**Age (years)**		**Male/Female**		**Time of start of intervention**
	**Experimental group**	**Control group**	**Experimental group**	**Control group**	
Meng et al. (22), Zhou et al. (28)	83.5 ± 2.3	84.2 ± 1.6	18/12	17/11	Symptomatic intracranial atherosclerotic stenosis
England et al. (23)	74.7 ±10.8	77.7 ± 10.4	4/9	5/8	Within 24h of AIS onset
Li et al. (24)	68.38 ± 6.76	64.32 ± 10.00	No description	No description	Within 72h of AIS onset
Che et al. (25)	66.1 ± 11.2	65.3 ± 9.4	11/4	13/2	Within 4.5h of AIS onset
England et al. (26)	70.9 ± 13.4	73.7 ± 10.2	21/10	15/14	Within 6h of AIS onset
Feng et al. (27)	64.16 ± 7.71	63.91 ± 7.61	28 /14	26 /18	Within 14 days of AIS onset
An et al. (29)	62.06 ± 12.1	67.09 ± 9.9	22/12	25/9	3h after intravenous thrombolysis
He et al. (30)	59.5 ± 8.5	61.3 ± 11.0	20/4	18/7	36–24 h after intravenous thrombolysis
Li et al. (31)	68.3 ± 5.47	66.7 ± 6.23	14/10	16/8	Within 3 days of AIS onset
Pico et al. (32)	67.8 ± 15.1	66.7 ± 16.4	45/48	53/42	Within 6 h of AIS onset
Poalelungi et al. (33)	66.78 ± 6.44	64.41 ± 9.02	11/7	13/9	Within 24 h of AIS onset

Data are presented as mean ± standard (SD); Number/number: Male/female. AIS, acute ischemic stroke.

### Evaluation of the quality of included studies

**Figure 2:**
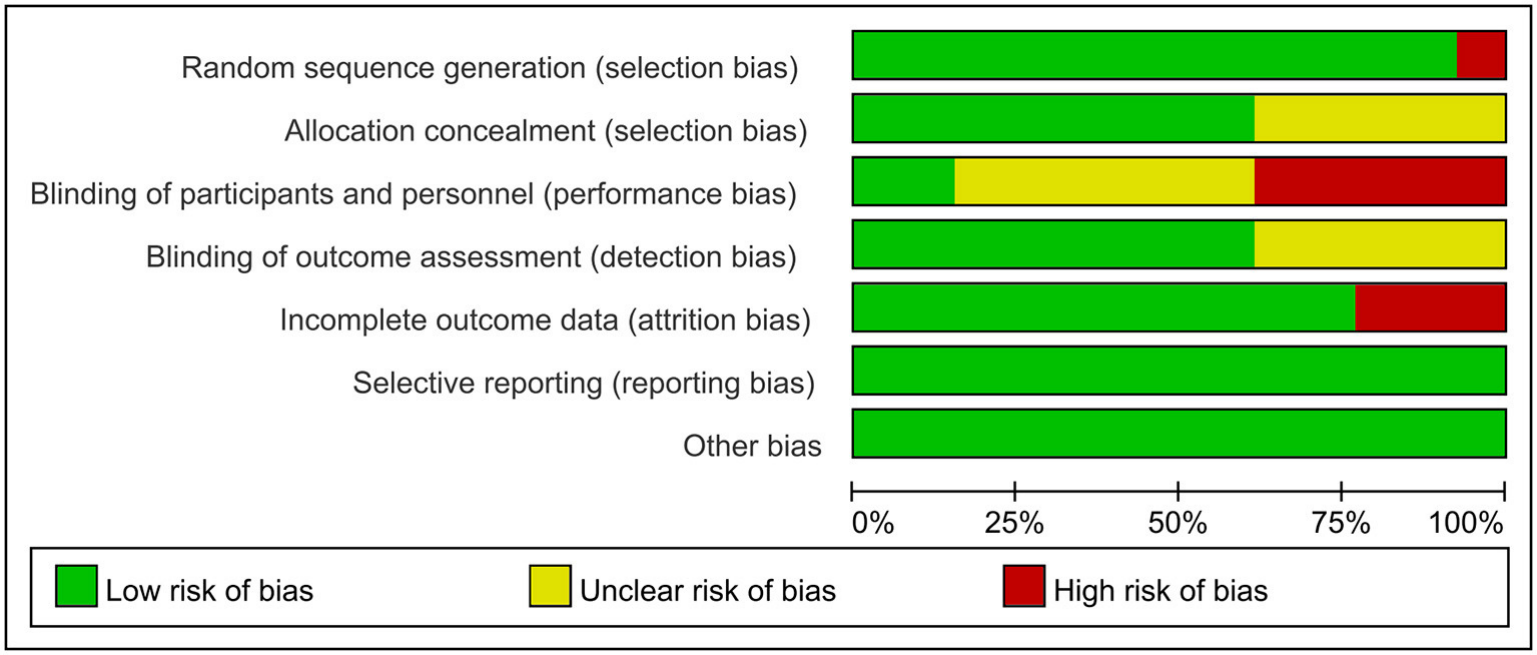
Evaluation of the quality of included studies.

**Figure 3:**
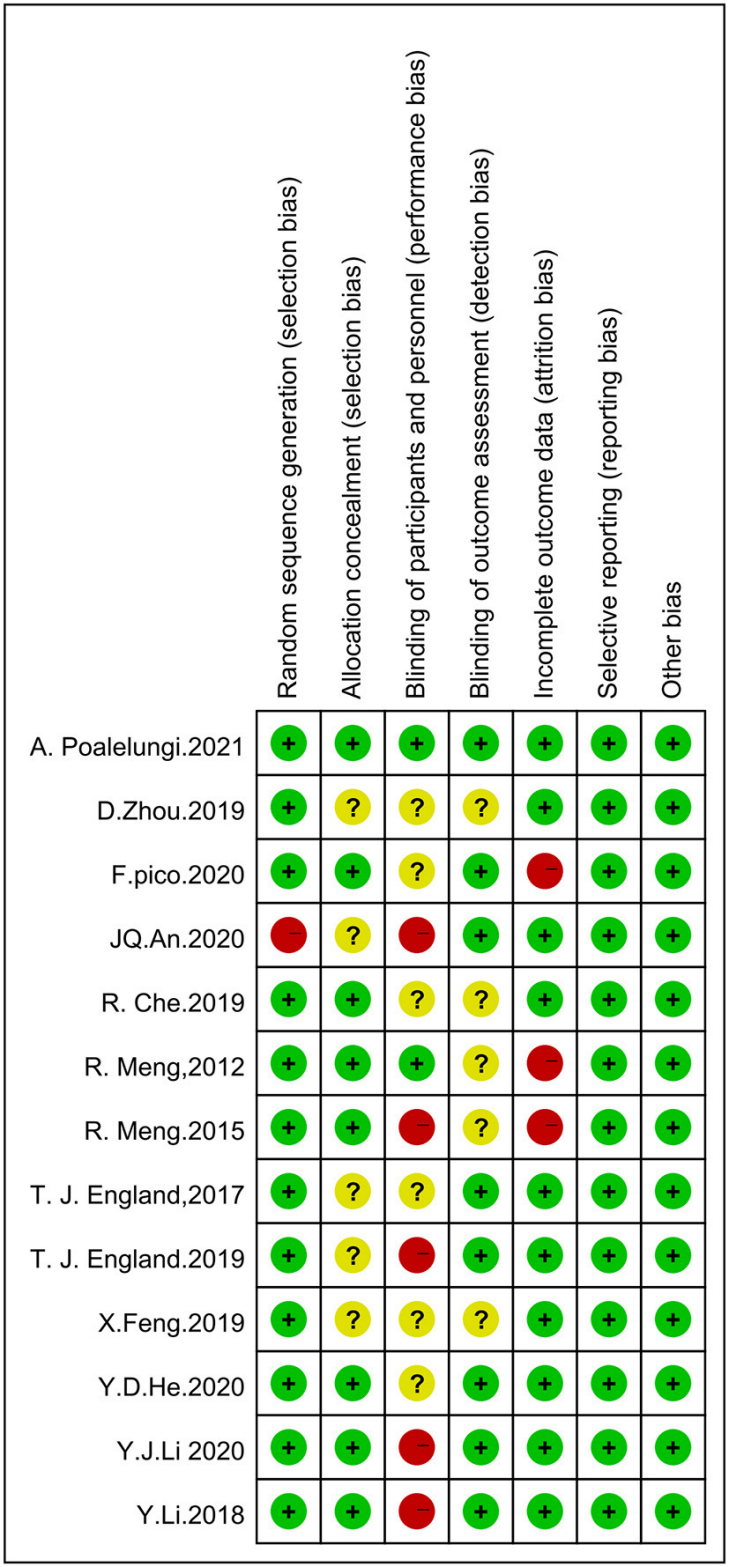
Evaluation of the quality of included studies.

Using the Cochrane Risk of Bias Tool to assess risk of bias, eleven studies reported using computerized randomization grouping and one study used patient ID tail numbers for randomization grouping; seven studies reported using envelopes to hide allocation sequences, four studies did not describe allocation hiding methods, and one study used a central network for allocation hiding; five studies reported that researchers could not be blinded, six studies did not report information on blinding of participants and study staff; eight studies reported blinding of outcome assessment and four studies did not report whether blinding was used for outcome assessment; ten studies reported complete data and two studies had >20% missing data; No selective reporting or other biases were found. The evaluation results are shown in Figures 2, 3.

### Statistical results

#### Effect of RIPostC on NIHSS scores

**Figure 4:**
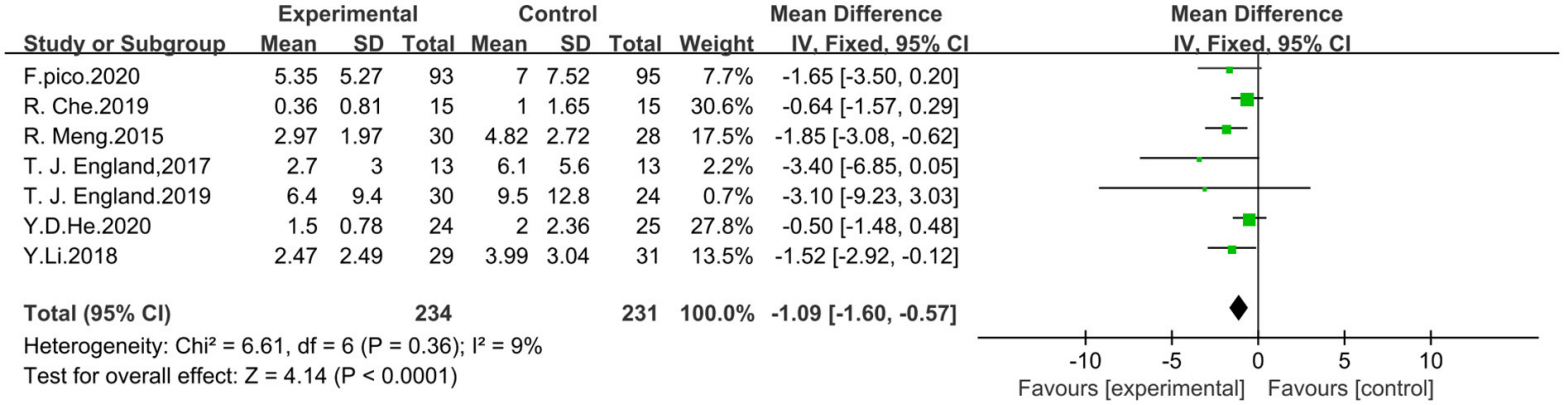
Forest plot showing effect of RIPostC on NIHSS scores.

Seven studies that included NIHSS scores were analyzed using fixed effect model for a total of 465 patients, 234 in the trial group and 231 in the control group. The meta-analysis showed that RIPostC significantly reduced NIHSS scores compared to the control group (MD: −1.09, 95% CI: −1.60, −0.57, *P* < 0.0001). The heterogeneity of included studies was not significant (heterogeneity: x^2^ = 6.61, I^2^ = 9%, *P* = 0.36). The forest map is presented in Figure 4.

#### Effect of RIPostC on the rate of good prognosis

**Figure 5:**
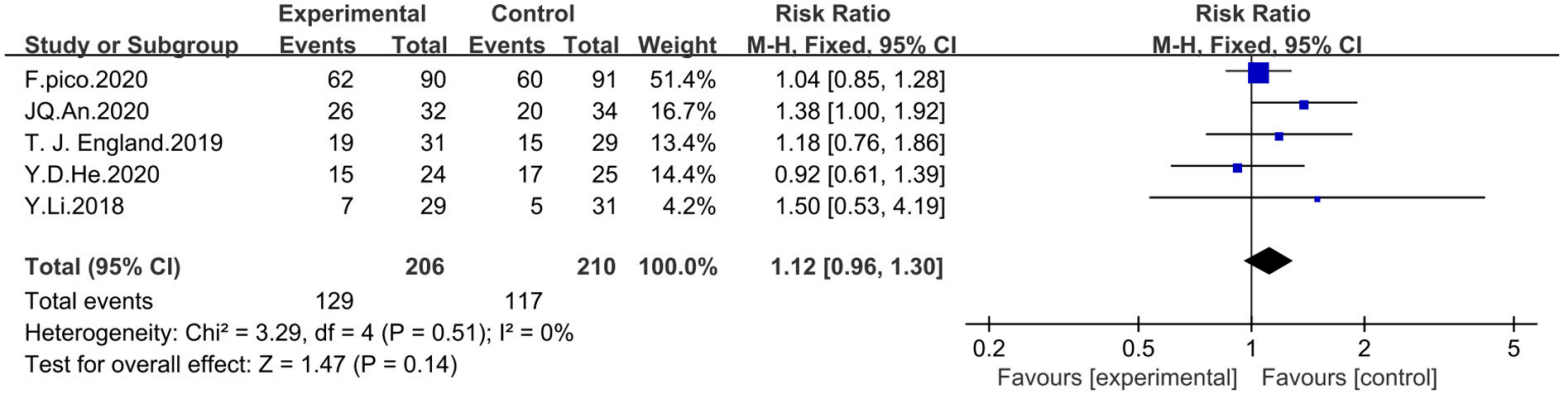
Forest plot showing effect of RIPostC on the rate of good prognosis.

Five studies incorporating mRs scores were analyzed by using fixed-effects model for a total of 416 patients, 206 in the experimental group and 210 in the control group. A score of mRs < 2 was considered to indicate good prognosis. The meta-analysis showed that RIPostC did not significantly improve the prognosis of patients with ischemic cerebrovascular disease compared with controls, based on a fixed-effect model (RR = 1.12, 95%CI: 0.96–1.30, *P* = 0.14). The heterogeneity of included studies was not significant (heterogeneity: x^2^ = 3.29, I^2^ = 0%, *P* = 0.51). The forest map is presented in Figure 5.

#### Effect of RIPostC on MocA scores

**Figure 6:**
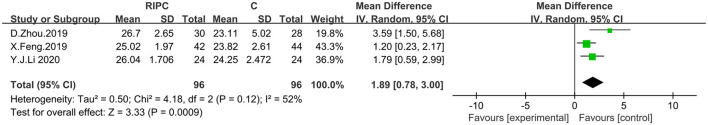
Forest plot showing effect of RIPostC on MocA scores.

Three studies incorporating the MoCA scores were analyzed by using a random-effects model with a total of 192 patients, 96 in the trial group and 96 in the control group. A meta-analysis showed that RIPostC improved cognitive function in patients, compared to controls, with ischemic cerebrovascular disease, based on a random-effects model (MD: 1.89, 95%CI: 0.78–3.00, *P* = 0.0009). Heterogeneity of included studies was significant, therefore a random effects model was used (x^2^ = 4.18, I^2^ = 52%, *P* = 0.12). By excluding the literature one by one, it was found that heterogeneity I^2^ = 0% when the study by Zhou et al. (28) was excluded. No cause of heterogeneity was found by comparing the three literatures. The forest map is presented in Figure 6.

#### Effect of RIPostC on Barthel scores

**Figure 7:**
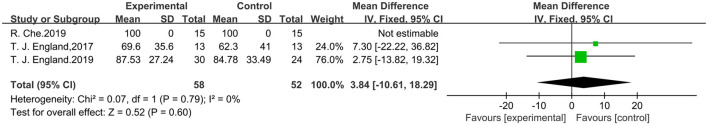
Forest plot showing Barthel scores.

Studies with three outcome indicators containing Barthel scores were analyzed using a fixed effects model. A total of 110 patients were included, 58 in the trial group and 52 in the control group. Meta-analysis showed that RIPostC did not significantly improve Barthel scores compared to controls, by using the fixed effects model (MD: 3.84, 95%CI: −10.61, 18.29, *P* = 0.60). Inclusion of study heterogeneity was not significant, so a fixed effects model was used with heterogeneity (x^2^ = 0.07, I^2^ = 0%, *P* = 0.79). The forest map is presented in Figure 7.

#### Security with RIPostC

**Figure 8:**
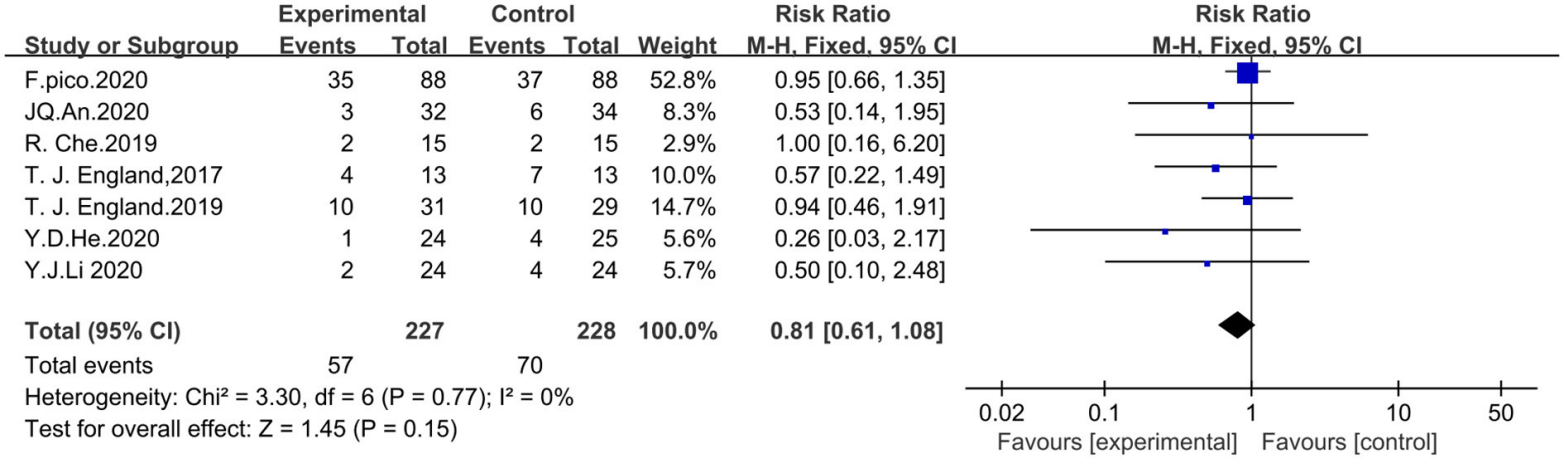
Forest plot showing security with RIPostC.

Seven studies that included the incidence of adverse events were analyzed with the fixed-effects model in which adverse events were defined as vascular events including cerebral hemorrhage, cerebral infarction, and transient ischemic attack. A total of 455 patients were included, including 227 in the experimental group and 228 in the control group. The meta-analysis showed that the difference in the incidence of adverse events between the RIPostC and control groups was not statistically significant (RR = 0.81, 95%CI: 0.61–1.08, *P* = 0.15). Inclusion of study heterogeneity was not significant, so a fixed effects model was used with heterogeneity (x^2^ = 3.30, I^2^ = 0%, *P* = 0.77). The forest map is presented in Figure 8.

#### Effect of RIPostC on changes in cerebral infarct volume

Three studies used cerebral infarct volume as the primary outcome indicator of the study, but only qualitative analysis could be done because quantitative synthesis was not possible. The cerebral infarct volume is measured in cm^3^ by a professional technician in combination with magnetic resonance imaging (MRI) or computed tomography (CT) and manually outlined using software. The outcome indicators in the three studies were the differences in the final cerebral infarct volumes between the RIPostC group and control groups at 24h (32), 90 days (24), and 180 days (33), respectively, and the results are shown in Table 4. The results of the study showed that the brain infarct volume in the RIPostC group was smaller than that in the control group after the intervention, but only the RIPostC group in Li et al. study (24) showed a significant decrease in brain infarct volume among the three studies. The other two studies did not show statistically significant differences, although the brain infarct volume in the RIPostC group was lower than that in the control group.

**Table 4:** Volume study characteristics of cerebral infarction.

**Research**	**Baseline**		**Post-intervention**		***P***
	**RIPostC group**	**Control group**	**RIPostC group**	**Control group**	
Li et al. (24)	8.62 ± 4.26	7.69 ± 7.37	3.5468 ± 2.06897	5.24632 ± 2.66309	< 0.05
Pico et al. (32)	9.3 (3.4, 38.3)	12.2 (3.7, 32.3)	13 (3.2, 54.7)	18.8 (4.9, 66.7)	NA
Poalelungi et al. (33)	10.16 ± 15.17	23.19 ± 43.02	9.38	10.35	0.4

Data are presented as mean ± standard (SD) or mean or median (interquartile range). NA, not available.

Poalelungi et al. (33) noted in the study that the reason for the difference not being statistically significant could be that the intervention had already been administered to the patients at the time of the baseline measurement, when RIPostC had already had an effect on the patients' cerebral infarct volumes. Further, in that study the baseline measurement was performed after the intervention had began; and another reason could be that the sample size was not large enough (*n* = 40). In the study of Pico et al. (32), there was almost no difference in the change in cerebral infarct volume between the two groups 24 h after the intervention. One reason may be that RIPostC was not effective in patients with untreated ischemic stroke because previous studies were combined with pharmacological treatment or reperfusion therapy, whereas this study was a RIPostC intervention when not treated prehospital, and another reason may be that in their study (32) the intervention was short, receiving only one RIPostC session at 6 h pre-hospital, while in the Li et al. (24) study, the RIPostC intervention lasted 14 days. However, it is also possible that RIPostC is not effective for patients and does not reduce the volume of cerebral infarction in patients with AIS.

### Publication bias

**Figure 9:**
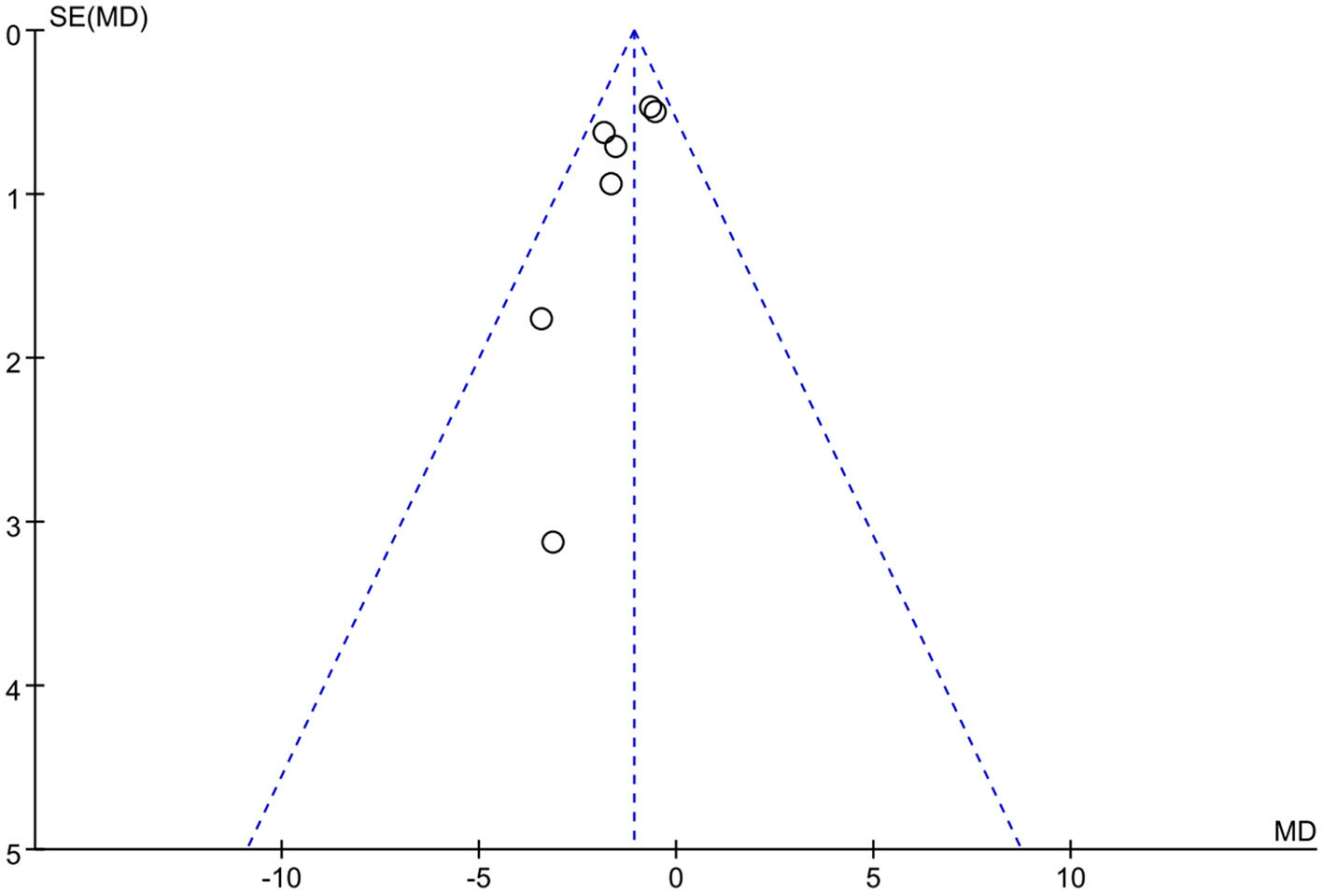
Funnel plot to detect risk of publication bias in the meta-analysis.

We used Rev Man 5.3 software to create funnel plots for the outcome indicators with the highest number of included studies, and the funnel plots revealed that the studies were largely symmetrically distributed and concentrated in the upper part of the funnel, so the possibility of publication bias was low. The funnel diagram is presented in Figure 9.

## Discussion

Ischemic post-conditioning has been shown to reduce IRI in several vital organs (5, 34, 35). It can protect the brain from IRI and improve neurological deficits through a variety of endogenous protective mechanisms. However, local post-conditioning is operationally difficult and risky, and cannot be applied on a large scale. The advent of RIPostC has furthered the clinical translation. As research has progressed, several animal studies have confirmed that RIPostC can improve the prognosis of cerebrovascular disease and can protect the brain from ischemic injury (13, 36, 37). Several potential mechanisms of action also explain the cerebroprotective effects of RIPostC, and studies have shown that RIPostC may mediate neuroprotection through glucagon-like-peptide-1 receptor (GLP-1R) activation (38) and may also exert cerebroprotective effects by mediating the release of extracellular vesicles (EVs) in the plasma (39).

As with the results of a previously published meta-analysis (40), the results of the current study showed that RIPostC significantly improved the degree of neurological deficits and reduced NIHSS scores in patients compared to controls. However, our meta-analysis showed that the RIPostC group did not significantly improve the prognosis of patients, and the difference in the rate of good prognosis between the two groups was not statistically significant, which is different from the results of the previous meta-analysis. The previous meta-analysis included less literature, and the current study included more literature, but the sample size is still not large enough. It is not reasonable to reject the effect of RIPostC based on this analysis alone, and more clinical studies to verify whether RIPostC can improve the long-term prognosis of patients should be added in the future. The current study also analyzed the effects of RIPostC on patients' cognitive function, self-care ability in daily life, and the safety of RIPostC. Our meta-analysis showed that RIPostC significantly improved cognitive function in patients with post-stroke cognitive impairment, and it was reported that RIPostC reduced central and peripheral glutamate levels in patients with ischemic cerebrovascular disease after ischemia-reperfusion (41), and elevated peripheral glutamate levels were associated with cognitive impairment in patients (42). However, RIPostC did not significantly improve patients' Barthel scores or their ability to care for themselves in daily life. It should be noted that this outcome indicator was included in only three studies with 110 patients and again may not yield statistically significant differences due to the small sample size. The difference in the incidence of adverse events between the two groups was not statistically significant compared to the control group, and therefore RIPostC was safe. With respect to the effect of RIPostC on changes in cerebral infarct volume, although animal studies have demonstrated that ischemic post-conditioning treatment reduces cerebral infarct volume (13), there are fewer clinical studies, and a clinical trial of remote ischemic per-conditioning before patients were admitted to hospital for treatment, which included patients with ischemic stroke, hemorrhagic stroke, and transient ischemic attack, and showed no statistical difference in final infarct size between the two groups (43). However, when adjusted for baseline severity of hypoperfusion, a voxel-byvoxel analysis demonstrated increased tissue survival after 1 month suggesting that prehospital remote post-conditioning may be neuroprotective. The risk of infarction and the degree of cytotoxic edema were lower in the RIPostC group than in the control group. Multiple clinical studies have failed to show that RIPostC can reduce cerebral infarct volume in patients, but as the most visual outcome indicator of AIS prognosis, it is critical to patient prognosis. Nevertheless, we still prefer the effectiveness of RIPostC because although there was no statistical difference, there was a decrease in final cerebral infarct volume in the RIPostC group compared with the control group, and a more standardized trial procedure with a larger sample size may prove the effectiveness of RIPostC in the future.

Standardization of intervention procedures is an important prerequisite for clinical translation of RIPostC. A comparison of the characteristics of the included studies revealed differences in the length of the intervention, and site of the intervention, the pressure used for the intervention, and the timing of the intervention. Li et al. (44) found that when performing multiple cycles of RIPostC training in rats, three cycles of RIPostC training were the best choice and showed better results than one, two, or four cycles. However, almost all clinical studies used four cycles of RIPostC training, and most of them proved to be safe and effective. If the effect of three cycles is better than that of four cycles, which can reduce the treatment time and at the same time improve patient compliance, future experiments can be designed accordingly to determine exactly how many cycles are optimum for RIPostC. The results of RIPostC in rhesus monkeys showed that multiple limb interventions are better than single limbs interventions (45). Are multiple limb interventions better than single limbs interventions in clinical applications? There are no clinical studies to comparing these two, and further research is warranted and research is still needed to explore. On the road to clinical translation of RIPostC, in addition to the need to identify interventional procedures, its potential protective mechanisms, risks, etc. need to continue to be explored by research (46). From the demographic data, it can be seen that most of the included patients' intervention start time is within 72h after the occurrence of AIS. Furthermore, owing to various reasons, many patients may not be able to receive the intervention within 72h. The clinical applicability of RIPostC is currently problematic, because it remains to be seen how and whether the intervention time window can be expanded and the means to simplify the intervention mode.

This paper has some limitations. First, the sample size included was small; although 12 studies were included, only one was a multicenter study, and the others were single-center, small sample studies and had small sample sizes, thus, the statistical differences may not be derived. In addition, the heterogeneity of the included studies was large, and the RIPostC intervention procedures varied among studies, mainly because there is no standardized RIPostC intervention procedure, and several studies are still needed to explore the best RIPostC intervention procedure in the future. Second, there are some ethnic differences in the populations of the included studies, given that most of the included studies were conducted in China. Therefore, it remains to be seen whether RIPostC has the same effect in patients from other regions.

## Conclusion

How to improve symptoms and reduce mortality and disability in patients with ischemic stroke has been an important direction of clinical research. The effect of RIPostC on ischemic cerebrovascular disease has been confirmed by several clinical studies, but because of the small number of clinical studies and the fact that some clinical studies have concluded that RIPostC is ineffective and therefore cannot be translated for clinical application, we conducted a meta-analysis to further analyze the effects and safety of RIPostC. In this meta-analysis, we quantified the effect of RIPostC on the prognosis of and disease severity in patients with ischemic cerebrovascular disease. Overall, RIPostC is safe and effective in patients with ischemic cerebrovascular disease, as it reduces the degree of neurological deficits and improves cognitive function, but meta-analysis showed that it did not significantly reduce the volume of cerebral infarction and improve the long-term prognosis of patients. More high-quality studies are needed in the future to explore the effectiveness of RIPostC and promote the clinical translation of RIPostC.

## Data availability statement

The raw data supporting the conclusions of this article will be made available by the authors, without undue reservation.

## Author contributions

ML, YW, and HL contributed to conception and design. ML and XY wrote the manuscript and figures, collected the data, and designed the figures. ML, YW, and YL performed quality evaluation and data extraction. All authors contributed to the article and approved the submitted version.

## Conflict of interest

The authors declare that the research was conducted in the absence of any commercial or financial relationships that could be construed as a potential conflict of interest.

## Publisher's note

All claims expressed in this article are solely those of the authors and do not necessarily represent those of their affiliated organizations, or those of the publisher, the editors and the reviewers. Any product that may be evaluated in this article, or claim that may be made by its manufacturer, is not guaranteed or endorsed by the publisher.
